# Video-Observed Therapy With a Notification System for Improving the Monitoring of Tuberculosis Treatment in Thailand: Usability Study

**DOI:** 10.2196/35994

**Published:** 2022-05-06

**Authors:** Ponlagrit Kumwichar, Virasakdi Chongsuvivatwong, Tagoon Prappre

**Affiliations:** 1 Department of Epidemiology Faculty of Medicine Prince of Songkla University Hat Yai, Songkhla Thailand

**Keywords:** app, compliance, usability, remote monitoring, therapy, tuberculosis, lung, infectious disease, user experience, video directly observed therapy, video-enhanced therapy, video-observed therapy, digital health, health care system, disease monitoring, health monitoring, video consultation, online health, virtual therapy

## Abstract

**Background:**

In Thailand, the health care system has struggled to cope with COVID-19, resulting in directly observed therapy for tuberculosis being de-emphasized. A video-observed therapy (VOT) system, or more specifically, the Thai VOT (TH VOT) system, was developed to replace directly observed therapy. According to the pilot study, the system needed notifications to improve usability and user compliance. The updated version of the TH VOT system thus enabled LINE (Line Corporation) notifications.

**Objective:**

This study aimed to reassess users’ compliance with and the usability of the updated TH VOT system.

**Methods:**

This study was conducted in the Hat Yai and Mueang Songkhla districts in Songkhla Province, Southern Thailand, from September 18 to December 1, 2021. The system was used by not only patients with tuberculosis but also tuberculosis staff, who acted as observers in primary health care settings. Some of the observers used the simulated VOT system instead of the actual system due to the lack of participating patients in their jurisdiction. After 30 days of using the system, VOT session records were analyzed to determine the compliance of the patients and observers. The User Experience Questionnaire was administered to reassess the usability of the system and compare the ratings of the participants with the general benchmark scores of the User Experience Questionnaire. The results were summarized to reveal the degree of user compliance and usability in the following three groups: the patients, actual VOT observers, and simulated VOT observers.

**Results:**

Of the 19 observers, 10 used the actual VOT system, and the remaining 9 used the simulated VOT system; there were also 10 patients with tuberculosis. The patients, actual VOT observers, and simulated VOT observers exhibited about 70%, 65%, and 50% compliance, respectively, in terms of following the standard operating procedures every day. The scores of all groups on all dimensions were well above the average scores. There was no significant difference in any of the dimensional scores among the three groups.

**Conclusions:**

The updated version of the TH VOT system was deemed usable by both the patients and the health care staff. Compliance with the use of the system was high among the patients but moderate among the observers.

## Introduction

Thailand—1 of 30 countries with the highest tuberculosis burden, especially multidrug-resistant tuberculosis burden [1]—has been hit hard by COVID-19 for 2 years. The health care system had struggled to cope with COVID-19, resulting in nonemergency services, including directly observed therapy (DOT) for tuberculosis, being de-emphasized [2,3]. This occurred in addition to DOT being widely criticized for its sloppiness [4-8]. Under this constraint, a video-observed therapy (VOT) system, or more specifically, the Thai VOT (TH VOT) system, was developed to replace DOT.

In our pilot study, the system was still not usable for observers in Na Yong District, Trang Province, Southern Thailand. Among the different dimensions of usability, stimulation, which is supposed to motivate users, was the least favorable. About half of the video sessions were sent and observed (37/70, 53%), indicating low compliance among the observers. In order to determine a solution, a qualitative study was conducted, and the observers suggested adding a notification system and an auditor to improve their observation tasks and compliance. The pilot study was also limited by an insufficient sample size and the short duration of users’ experience [9].

Based on the aforementioned suggestions, an improved version of the TH VOT system was developed. The updated system was integrated into the DOT programs of primary care units (PCUs) in the Hat Yai and Mueang Songkhla districts in Songkhla Province, Southern Thailand. The tuberculosis staff of PCUs were the observers who monitored the patients in their juristic areas. One auditor exclusively evaluated the observers and gave feedback. This study aimed to reassess users’ compliance with and the usability of the updated TH VOT system.

## Methods

### Enabling a Notification System

The LINE (Line Corporation) notification application programming interface had been created as part of our previous study [9]. However, the notification function was disabled at that time for reasons of cost reduction. The LINE notification system is common in Thailand; therefore, the notification function was enabled to improve the user experience dimension of simulation [10].

### Details of the LINE Notification System Added in the Standard Operating Procedures

**Figure 1:**
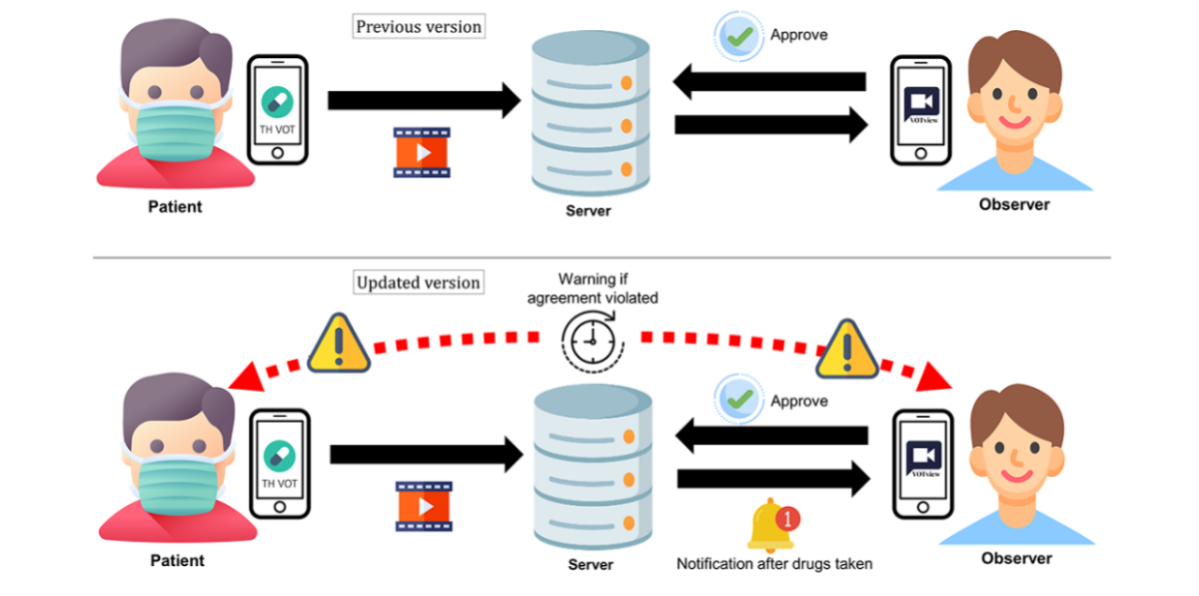
A comparison of the previous and improved versions of the TH VOT system. TH VOT: Thai video-observed therapy.

Details were added to the standard operating procedures (SOPs) [9] for the previous version of the TH VOT system with regard to the notification system. The time to take medication was determined and revised, if necessary, by the patients. The LINE notification would pop up on the patients’ smartphones if they did not record a video by using the TH VOT app within 30 minutes of the time set for taking medication. The notification alarm would sound immediately after a patient sent a video. Moreover, the notification would also notify an observer to remind the patients to take their medication if this was not done within a predetermined time interval. The system notification would also send a secure link to the observers, which led to the lists of patients under their jurisdictions who did not send a video. The observer could then call the patients by using the “call” buttons on the lists. The auditor would then conduct a daily evaluation of the VOT sessions of the patients and their observers by using the electronic database and judge whether the sessions were completed properly. Feedback on individual performance was then sent to users every weekend. Hence, the notifications for both the patients and the observers could enhance the system, making it more efficient, accurate, and stimulating than the previous version (Figure 1).

### Implementation of the Updated TH VOT System

To obtain a larger sample size of users, the study site was moved to the Hat Yai and Mueang Songkhla districts in Songkhla Province, which has the highest tuberculosis burden in Southern Thailand. The sample size was estimated by using 1 mean sample formula [11] and compared with the general benchmark to test whether the simulation score was above the “good” level (>1.35) [12]. We estimated that the mean simulation score of the User Experience Questionnaire (UEQ) for the observers would be 2 (it was 0.67 in the pilot study), while the SD of the mean score was estimated to be 1. Other aspects of the settings were similar to those of the pilot study’s setting. As there were several PCUs in the study area, 9 and 10 PCUs from Hat Yai and Mueang Songkhla districts, respectively, were randomly selected as the sites of implementation. For each PCU, 1 tuberculosis staff member was assigned to be an observer. Thus, at least 19 observers were required. The number of patients for each observer was flexible, depending on how many patients they could invite for usability testing.

The 19 recruited tuberculosis staff, as the observers, familiarized themselves with the system by following the training that was described previously in the pilot study [9]. In brief, the use of the TH VOT system was explained and demonstrated to the observers. After the training, each observer was assigned to invite and train the patients with tuberculosis in their jurisdictions by using the TH VOT app. This secondary training step was supervised and approved by the researchers before the actual VOT process was launched. Only patients with pulmonary tuberculosis who were treated during the continuous phase of tuberculosis treatment and had a smartphone were eligible for inclusion. The patients who were scheduled to complete treatment within 30 days were excluded. The recruited patients were scheduled to take medication once per day for 30 days, and they received cellular internet support for using the TH VOT app to record and send a daily video to their observers for 30 days. Each observer had to approve the videos sent by their patients within 24 hours. If an observer could not invite any eligible patient with tuberculosis, the videos that had been recorded in the pilot study would be used instead as part of the simulated VOT system for usability testing. The patients in the videos had consented to being observed by tuberculosis staff in other areas for training purposes. For the observers without patients, daily notifications would be randomly sent without a video 3 days per week to indicate that the automated system inconstantly sent videos like the actual VOT system. On those days, each observer had to press the “call” button in the notification link to remind the patients to send a video. The changes that were made to the system from the pilot study to create the current system are presented in Table 1.

**Table 1:** Comparison of this study’s setting and the pilot study’s setting.

	Pilot study	This study
Sites	Nayong District, Trang Province	Hat Yai and Mueang Songkhla districts, Songkhla Province
Centers	3 primary care units	19 primary care units
Patients	Real patients with tuberculosis who were treated with an isoniazid-rifampicin regimen and had a smartphone or simulated patients who had a smartphone (village health volunteer)	Real patients with tuberculosis who were treated with an isoniazid-rifampicin regimen and had a smartphone or a simulated video-observed therapy system (automatic video sending)
Observers	Tuberculosis staff from the primary care units were trained with the original instructions	Tuberculosis staff from the primary care units were trained with the updated instructions
Auditor	None	1 auditor for all
Video-observed therapy system	No notification system	LINE notification system added
Duration of observation	14 days	30 days

### Ethics Approval

This study was approved by the Human Research Ethics Committee, Faculty of Medicine, Prince of Songkla University (approval number: 64-03618-9). The researchers and deputy province chief medical officer of the Songkhla Provincial Public Health Office came to an agreement for the implementation of the TH VOT system.

### Compliance and Usability Assessment

#### Study Design

A prospective study was conducted among the aforementioned 19 observers and their patients with tuberculosis. The participants were recruited from September 18 to November 1, 2021. The staff were trained as observers from September 18 to 30, 2021. Then, the staff using the actual VOT system and their patients were trained together and certified by the researchers on November 1, 2021. They were then assigned to use the TH VOT system from November 1 to 30, 2021. A daily VOT session, which was comprised of the patients who were properly taking medication, was recorded in 1 video that was sent to the observer and approved within 24 hours. Then, the compliance of the users was judged by the auditor. Usability was assessed by patients and observers through the UEQ [10].

#### Data Collection

**Figure 2:**
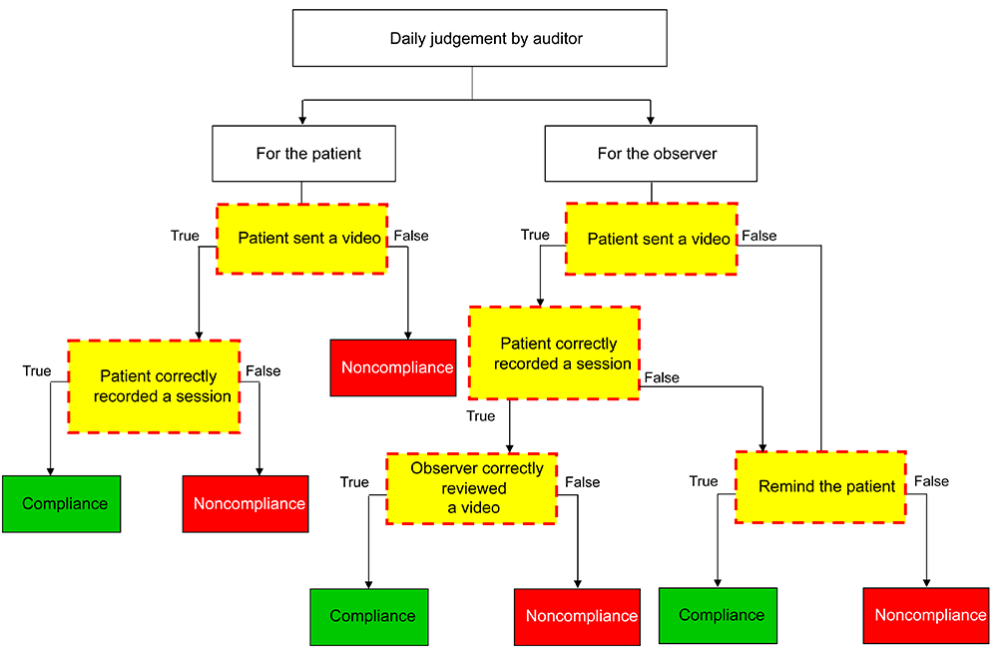
Algorithm of the auditor’s judgment.

On each day of the study period, the auditor assessed both the patients’ performance and the observers’ performance by using the SOP. Their assessment was recorded in a system that also contained all of the VOT data, as shown in Figure 2.

#### The SOP for the Assessment of Patient and Observer Compliance by the Auditor

The time unit for the judgment of compliance was “day,” and local times (Greenwich Mean Time + 7 hours) were recorded. The morning began at 12 AM (midnight), and the evening ended at 11:59 PM. However, daily compliance was judged as “achieved” if the patients took their medication and sent their videos before 6 AM the next day. The observer was considered compliant if they reviewed the videos within 24 hours after the videos had been sent. Therefore, the day that compliance was judged for the observer was always 1 day behind that for the patients.

A patient was only considered compliant if their video was sent before the cutoff and the quality of the video passed the following criteria:

1. The patient’s face and the drug tablets or capsules were clearly visible in the video frame.
2. The pills were picked up from the drug plane and put on the tongue.
3. The pills were swallowed along with clear water from a (clear) glass.
4. The tongue was then raised to show the sublingual area and stuck out to show the palate area.
5. Steps 2 to 4 were repeated until all of the pills were completely taken.

The observer was only considered compliant if they did the following to a quality standard: (1) assess whether the patients performed the aforementioned steps, (2) note the number of pills taken by the patient, and (3) make a reminder call if the patients did not send a video or incorrectly performed any procedure.

All daily VOT session records were automatically registered in the database of the TH VOT system, but only the usernames, dates, and times of the session records, which were confirmed by the auditor, were retrieved for compliance analysis. After 1 month of using the system, all users were administered the validated Thai UEQ [13]. It contained the following six dimensional scales: attractiveness (6 items), perspicuity (4 items), efficiency (4 items), dependability (4 items), stimulation (4 items), and novelty (4 items). The items were developed based on a semantic differential scale ranging from −3 to 3. In this scale, positive terms are given a score of 1, 2, or 3; neutral terms are given a score of 0; and negative terms are given a score of −1, −2, or −3 [10]. All participants were assured that they would be eligible to receive a reward regardless of how they answered the questions. Further, 1 UEQ sheet was sent to each participant by a delivery person in the first week of system use. The participants scored the TH VOT system (ie, via the UEQ) at home and sent the UEQ sheet back to the delivery person at the end of the observation phase. The delivery person put all of the sheets into a concealed box and gave it to the researchers. The UEQ sheets had no identification numbers and only indicated whether a respondent was a patient, an actual VOT observer, or a simulated VOT observer. No one could identify who provided particular responses. The mean UEQ scores of the six dimensional scales were compared with the average and good general benchmark scores [12].

#### Data Analysis

The users were categorized into the following three groups: patients, actual VOT observers, and simulated VOT observers. The basic characteristics of the users were summarized by using descriptive statistics. For each group of users, the average number of VOT sessions completed within 30 days by a user was estimated by using the mean cumulative function (MCF) of the Nelson-Aalen estimator [14]. The 95% CIs of the MCFs were produced via bootstrapping with 1000 replicates from resampling procedures. Ideally, the MCF value would be 30 if all participants performed sessions every day for 30 days. The mean UEQ scores were aggregated into 4 groups—the UEQ scores of the aforementioned groups of users and those of the overall observers. The UEQ scores were then visualized by using violin plots [15], in which mean scores and SEs were compared against the benchmark line. If the lower limit of the SE was over the reference line, it was determined that the corresponding UEQ score was significantly above the reference scores.

## Results

Of the 19 observers, 10 had a participating patient, while the rest of them had no patients and used the simulated VOT system instead. Table 2 summarizes the characteristics of the observers and patients and their VOT-related behaviors. The mean ages of the patients and observers were approximately 50 and 37 years, respectively. The basic characteristics of the observers for the actual and simulated VOT systems were similar. All patients performed VOT in the evening or after midnight and spent about 1 minute recording and sending their videos. The time between the patients’ and the observers’ sessions varied considerably because different observers viewed the videos at different times. The average time spent reviewing the videos was approximately 1.5 minutes. The notifications for the simulated VOT system were sent to the observers regularly (every day at 7 PM), and the observers responded to the videos shortly after their receipt (no more than 4 hours after receiving the videos). Only videos that showed complete sessions were sent to the observers for the simulated VOT system; thus, their reviews were completed faster (within 1.2 minutes) than those of the observers for the actual VOT system.

**Figure 3:**
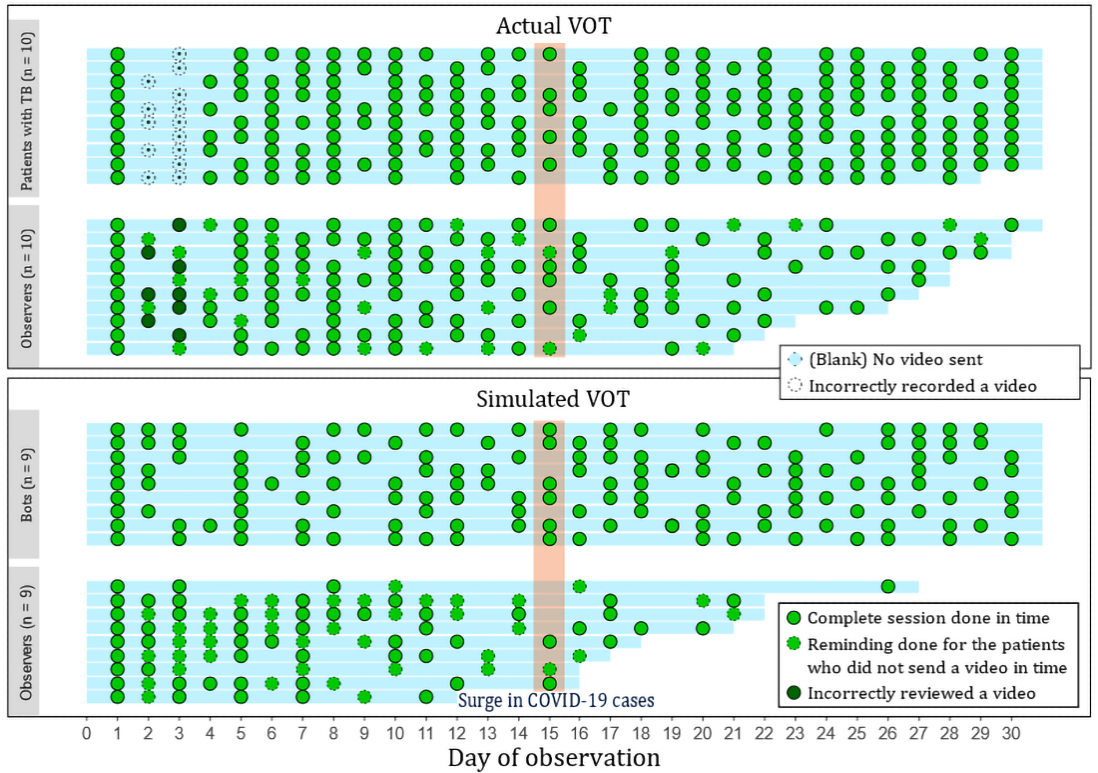
Visualization of the VOT sessions completed by the three groups of users (solid dots). TB: tuberculosis; VOT: video-observed therapy.

Table 3 summarizes descriptive statistics regarding participant compliance over a 30-day period for the three groups. The level of compliance was higher among the patients than that among the observers. Differences in compliance are visualized in Figure 3. Participants' daily compliance, as judged by the auditor, is represented by a dot for each day. The results show that the observers who used the actual and simulated VOT systems gradually disregarded their observation tasks and eventually became noncompliant with the protocol. On the first day, all participants were trained in accordance with the standard instructions [9] and performed a real session correctly. On the second and third days, all patients forgot to show their palate area; thus, their observers were not able to adequately see their compliance. The auditor therefore made calls to remind all participants to not miss this step. Among the 72 instances (patient days) when patients forgot to send a video, there were 32 instances (44%) in which they were reminded by the observers. For the simulated VOT system, observers sent reminders on 40 out of the 123 (32.5%) “bot days” that required a call action. During the study period, there was 1 festival day—*Loy Kratong*—on November 19, 2021, but it did not affect compliance. The days of noncompliance appeared to be random and did not follow a particular pattern for the patients with tuberculosis and actual VOT observers. For the simulated VOT observers, their compliance dramatically dropped due to the COVID-19 situation after November 15, 2021. After this point, the compliance of the simulated VOT observers (who were also tuberculosis staff) diminished, as they were needed to help control SARS-CoV-2 infection due to a lack of health care workers. Although the number of actual VOT observers did not suddenly diminish, it gradually decreased over time.

The 30-day MCFs in the patient, actual VOT observer, and simulated VOT observer groups were 21.79, 19.03, and 14.65, respectively, indicating that the three groups exhibited about 70%, 65%, and 50% compliance, respectively.

**Figure 4:**
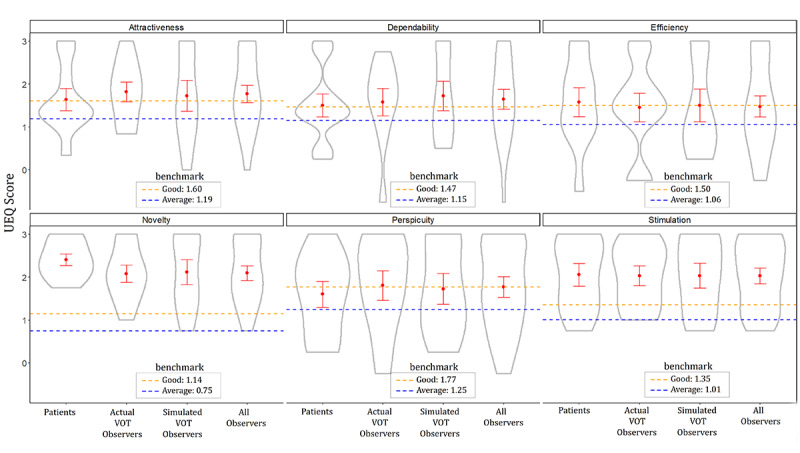
The six dimensions of UEQ assessed by the users within 30 days. UEQ: User Experience Questionnaire; VOT: video-observed therapy.

Figure 4 depicts the violin plots for each group of users and each dimension of the UEQ. The summation of UEQ scores could range from −18 to 18; however, each participant’s scores ranged from 3.58 to 18. The mean of the sum score was 11.89 (SD 3.99), and there were no outliers. The means and SEs are also presented within the violin plots as dots and error bars, respectively. The dotted horizontal lines for each dimension denote a good score (orange) and the average score (blue), which were compared to the general benchmark [12]. The scores of all groups on all dimensions were well above the average scores. Only the scores for the novelty and stimulation dimensions were significantly above the good scores. There was no significant difference in any of the dimensional scores among the three groups and overall observers.

**Table 2:** Participants’ characteristics and their video-observed therapy (VOT) behaviors.

Characteristics and behaviors					Actual VOT (users: n=20)		Simulated VOT (users: n=9)		*P* value^a^
**Patient characteristics and behaviors (n=10)**									
	**Sex, n (%)**								—^b^
		Female		4 (40)		—			
		Male		6 (60)		—			
	Age (years), median (SD)		50.6 (13.6)			—		—	
	Appointment time, median (minimum, maximum)		8 PM (7 PM to 9 PM)			7 PM (7 PM, 7 PM)		—	
	Lapse time between video appointment and uploading a video (minutes), median (minimum, maximum)		230.20 (104.4, 260.9)			0 (0, 0)		—	
	**Uploading a video daily, n (%)**								<.001
		Within 1 day		217 (72)		270 (100)			
		After midnight		83 (28)		0 (0)			
	Upload time within 1 day, median (minimum, maximum)		9:01 PM (7:29 PM, 11:59 PM)			7 PM (7 PM, 7 PM)		—	
	Upload time after midnight, median (minimum, maximum)		1:13 AM (12:02 AM, 2:01 AM)			—		—	
	Duration of recording a video (seconds), median (IQR)		56.3 (47.5-66.4)			59.6 (50.3-65.8)		.13	
**Observer characteristics and behaviors (n=19)**									
	**Sex, n (%)**								.99
		Female		6 (60)		5 (56)			
		Male		4 (40)		4 (44)			
	Age (years), mean (SD)		35.9 (4.6)			39.4 (4.8)		.12	
	Lapse time between video uploading and reviewing (minutes), median (IQR)		322.6 (156.8-678.1)			81.9 (71.6-121.3)		.008	
	Review time, median (minimum, maximum)		3:46 PM (7:42 AM, 10:51 PM)			8:15 PM (7:02 PM, 10:45 PM)		—	
	Duration of reviewing a video (seconds), median (IQR)		102.7 (89.5-119.5)			79.4 (70.6-88.9)		<.001	

^a^*P* values were calculated by using a Fisher exact test for all categorical variables, a 2-tailed Student *t* test for age, and a Wilcoxon rank-sum test for the duration of recording and reviewing.

^b^Not available.

**Table 3:** Average compliance in video-observed therapy (VOT) sessions completed by a user within 30 days (30-day mean cumulative function [MCF]) for each group.

Participants		Average session compliance of users within 30 days, MCF	Compliance, %^a^ (95% CI)
Patients (n=10)		21.79	72.6 (70.0-75.2)
**Observers**			
	Actual VOT (n=10)	19.03	63.4 (60.6-66.3)
	Simulated VOT (n=9)	14.65	48.8 (44.2-53.5)

^a^Percent compliance was calculated as follows: MCF/30 × 100.

## Discussion

### Principal Findings

In the revised version of the TH VOT system, we added a notification and audit system to improve the accountability of users. This study reassessed the usability of the revised version of the system. The usability assessment, which was performed with the UEQ, showed that the system was usable at an above-average level for all dimensions. Good stimulation—the most important aspect in sustaining medication adherence—was achieved. In the actual VOT system, there were no particular patterns in the days that the patients and observers achieved compliance and noncompliance. Among the three groups of users, the patients almost reached the highest amount of compliance (70%). The average level of compliance across all three groups was above 50%. Compliance was easier to achieve for the simulated VOT observers because the simulated VOT videos were sent at a set time each day, as opposed to the actual VOT observers who received patients’ videos at varying times. However, the simulated VOT observers’ compliance levels sharply dropped when they had other responsibilities (ie, helping to control COVID-19). This did not seem to immediately affect the compliance of the actual VOT system observers, as they tried to continue their observation tasks for the sake of the patients with tuberculosis. However, their compliance did gradually dwindle over time owing to an increase in the number of COVID-19 cases. This was mirrored in many other countries where the level of tuberculosis care decreased due to the burden of COVID-19 [8,16,17].

In comparison to the previous version [9], the improved TH VOT system appeared to improve the compliance of both patients and observers via notifications and audits. The instructions regarding compliance protocols for VOT were meticulously developed to ensure that observers and patients completed all of the necessary steps. For patients, there was a learning curve for maintaining compliance because of the complexity of instructions, as evidenced by the noncompliant videos that were sent on the first few days. However, as time went by, the patients’ compliance was able to be kept at a reasonable level.

Unlike previous studies in the United States and United Kingdom [18,19], the observers in our study context (Thailand) could not be laypeople because of privacy concerns. Due to the shortage of paramedics in PCUs during the COVID-19 pandemic, the staff (observers) had an increased workload, and the traditional DOT program was almost completely ignored. In addition to routine work, the tuberculosis staff were responsible for many other tasks in their PCUs.

According to our previous study [9], all tuberculosis staff said that at best, they could perform DOT twice per month. With its feasible features and audit system, the TH VOT system has the potential to improve tuberculosis treatment monitoring among tuberculosis staff by increasing compliance with the DOT program from almost 0% to at least 50%. If the COVID-19 surge had not taken place, we would have been able to better assess compliance.

The number of participants in this study was too small to be able to evaluate the effectiveness of VOT on tuberculosis treatment outcomes. Hence, a larger-scale study that compares traditional DOT and VOT is needed. In addition, this study did not include a control group, and no medical outcome was assessed; thus, a randomized control trial (VOT vs DOT) is needed.

### Conclusion

The updated version of the TH VOT system was considered usable by both the patients and the health care staff. Compliance with the use of the system was high among the patients (about 70%) and moderate among the observers (about 50%-65%). In this study, the reported usability scores showed that the TH VOT system was acceptable. Based on the unanimous above-average scores for all dimensions, we suggest that the system should be studied further and does not need any major changes. A randomized control trial should be conducted to ensure the effectiveness of the TH VOT system for tuberculosis treatment.

## Abbreviations

DOT: directly observed therapy
MCF: mean cumulative function
PCU: primary care unit
SOP: standard operating procedure
TH VOT: Thai video-observed therapy
UEQ: User Experience Questionnaire
VOT: video-observed therapy
